# Comparative Analysis of Cadmium Profiles in Liver, Kidney, and Muscle of Bovines, Equines, and Cervids

**DOI:** 10.3390/toxics14090806

**Published:** 2026-09-10

**Authors:** Stefania Squadrone, Alessandra Griglione, Paolo Palmegiano, Stefania Gavinelli, Anna Riva, Leo Costa, Diana Giuffrida, Marcello Gigliotti, Chiara Marchese, Rosa Avolio, Maria Cesarina Abete

**Affiliations:** Istituto Zooprofilattico Sperimentale del Piemonte, Liguria e Valle d’Aosta, via Bologna 148, 10154 Turin, Italy

**Keywords:** ICP-MS, Principal Component Analysis, food safety, environmental biomonitoring, bioaccumulation, veterinary public health

## Abstract

Understanding interspecies physiological variability, toxicokinetic pathways, and bioaccumulation dynamics is fundamentally essential for veterinary diagnostics, food safety assurance, and wildlife conservation management. This ten-year toxicological study (2015–2025) analyzed cadmium (Cd) concentrations in liver, kidney, and muscle tissues of bovines, cervids, and equines from northwestern Italy using ICP-MS. Cadmium distribution varied significantly by species and tissue. Bovines and cervids exhibited low, homogeneous burdens (bovine mean liver: 0.14 mg/kg, kidney: 0.60 mg/kg; cervid mean liver: 0.13 mg/kg, kidney: 0.72 mg/kg), and cervids showed wider ranges due to environmental exposure. Conversely, equines accumulated drastically higher concentrations in hepatorenal tissues, averaging 10 mg/kg in the liver and 53 mg/kg in the kidney. Muscle tissue concentrations remained minimal across all species (0.017 to 0.078 mg/kg). Principal Component Analysis explained 90.4% of total variance, cleanly separating equines from bovines and cervids based on hepatic and renal loads. Liver and kidney proved to be robust biomarkers for monitoring Cd exposure. These findings support the integration of comparative tissue metal profiling into comprehensive veterinary toxicology, veterinary public health, and wildlife health assessment frameworks.

## 1. Introduction

Evaluating how different species function physiologically, store or neutralize toxins, and process chemicals over time is fundamental to environmental monitoring, veterinary toxicology, food safety, and wildlife health protection. Cadmium (Cd) is a non-essential toxic transition metal characterized by high environmental persistence, an exceptionally long biological half-life spanning decades in mammalian systems, and a marked physiological tendency to accumulate in metabolically active organs, particularly the liver and kidneys [1]. As a ubiquitous environmental pollutant, cadmium originates from both natural geophysical weathering processes and extensive anthropogenic activities, including metallurgical industries, fossil fuel combustion, phosphate-based mineral fertilizers, sewage sludge disposal, and intense vehicular traffic deposition. Cd is categorized as a Group 1 human carcinogen by the International Agency for Research on Cancer [2]. Being persistent, Cd is widely distributed in terrestrial ecosystems; following environmental exposure, this element can accumulate in animal tissues through contaminated feed, water, and forage, with the liver and kidneys representing the primary target organs due to their central role in metal metabolism, systemic transport, and detoxification [3].

At the molecular and cellular levels, chronic cadmium exposure induces the intracellular generation of reactive oxygen species (ROS), including hydroxyl radicals, superoxide anions, and hydrogen peroxide, leading to severe lipid peroxidation, structural protein oxidation, DNA strand breakage, and the progressive depletion of vital cellular antioxidant defences such as reduced glutathione (GSH) and metalloenzymes [4]. Chronic Cd accumulation has been consistently associated with oxidative stress, disruption of trace-element homeostasis (competing with essential divalent cations such as zinc (${\mathrm{Zn}}^{2+}$), copper (${\mathrm{Cu}}^{2+}$), and calcium (${\mathrm{Ca}}^{2+}$) via molecular mimicry), and progressive impairment of hepatic and renal organ function [4].

Because cadmium persists for a long time and accumulates up the food chain, it poses a major dietary concern; consuming tissues from livestock or wild game can significantly increase human exposure and long-term health risks [5]. Since Cd uptake, systemic distribution, retention, and excretion kinetics are heavily influenced by species-specific physiological, anatomical, metabolic, and ecological factors, significant differences occur even among diverse animal populations inhabiting similar geographical environments [6]. Domestic and wild ungulates are widely utilized globally as bioindicators of environmental contamination because their internal tissue metal burdens reliably reflect regional exposure levels and food chain transfer dynamics [7]. Cattle generally exhibit relatively homogeneous exposure patterns associated with controlled intensive or semi-intensive feeding systems and regulated dietary inputs, whereas free-ranging cervids may reflect a much broader range of environmental conditions, seasonal dietary shifts, altitudinal foraging variations, and localized soil–plant transfer rates [8].

Equines represent a uniquely fascinating physiological and toxicological model due to their extended longevity, specialized digestive physiology as hindgut fermenters (which alter gastrointestinal transit times and absorptive surface interactions), and a recognized extraordinary capacity to sequester and accumulate trace elements over extended developmental and chronological periods [9]. In long-lived mammals, specific intracellular proteins, metallothioneins (MTs), mediate systemic metal homeostasis, cellular detoxification, and longevity-associated stress responses [10]. MTs are low-molecular-weight, cysteine-rich proteins that bind cadmium with extremely high affinity, effectively neutralizing its free ionic toxicity within hepatocytes and renal proximal tubular cells. However, while initial sequestration protects against acute cytotoxicity, the continuous renal and hepatic accumulation of metal–protein complexes over decades underpins the extreme terminal burdens observed in older equines [10].

Although Cd accumulation has been extensively investigated in single animal species, comparative studies evaluating species-specific patterns of tissue distribution across multiple ungulate groups remain remarkably limited in scientific literature. Multivariate statistical approaches offer exceptionally valuable chemometric tools for exploring complex biological variability, reducing data dimensionality, and identifying organ-specific accumulation signatures that may not be apparent through conventional univariate analyses. Principal Component Analysis (PCA) enables the simultaneous evaluation of multiple tissue compartments, facilitating the rapid identification of underlying patterns associated with species-specific physiological responses and toxicokinetic capacities.

The present study aimed to comprehensively compare Cd concentrations in the liver, kidney, and muscle tissues of bovines, equines, and cervids from northwestern Italy, and to investigate whether tissue accumulation profiles could reliably discriminate among these distinct species. We hypothesized that hepatic and renal Cd burdens would represent the principal statistical determinants of interspecies variability and could serve as robust, validated biomarkers of species-specific metal accumulation.

Additionally, this study evaluates these metal profiles against current European Union food safety regulations to provide veterinary public health authorities with practical data for decision-making.

## 2. Materials and Methods

### 2.1. Species

Bovines (*Bos taurus* Linnaeus, 1758) are domestic ruminants that consume managed, relatively uniform diets consisting of pastures and formulated feed. As ruminants with a multi-chambered forestomach and slower fermentation kinetics, their digestive system alters how metals are released from food matrices, influencing bio accessibility and absorption rates across the intestinal wall.

Equines (*Equus ferus* caballus Linnaeus, 1758) are monogastric hindgut fermenters featuring a rapid rate of passage for digesta. They typically have a much longer natural lifespan and higher slaughter age compared to ruminants, which provides an extended chronological window for hepatic and renal metal bioaccumulation across their liver, kidney, and muscle tissues.

Cervids (*Cervus elaphus* Linnaeus, 1758) are wild ruminants that act as selective browsers feeding on twigs, bark, leaves, and fungi. Like bovines, their multi-chambered forestomach and slower, more intensive fermentation kinetics influence the gastrointestinal environment, bio accessibility, and intestinal absorption rates of heavy metals.

### 2.2. Sample Preparation

A total of 270 biological samples comprising liver, kidney, and muscle tissues (90 samples per tissue type, evenly distributed across three species with 30 adult individuals per species) from bovines, equines, and cervids originating from northwestern Italy were collected and analysed for total cadmium content.

The final sample size was naturally constrained by the availability of equine specimens, which yielded a total of 30 samples. To maintain a balanced experimental design and avoid the statistical limitations associated with severely unequal group sizes, the sample size was standardized to 30 for all groups. For bovines and cervids that had larger available pools, a subset of 30 individuals was selected from the larger dataset using Microsoft Excel. Specifically, a random number was assigned to each row, the dataset was sorted randomly based on these values, and the first 30 rows were selected. This automated, objective procedure ensured that the selection was entirely independent of any phenotypic or experimental characteristics, completely preventing systematic bias.

Specimens were collected following regional veterinary hygiene regulations, ensuring samples came from healthy adult cohorts representing standard slaughterhouse throughput. All samples were collected in the frame of official veterinary control activities and targeted regional monitoring programs from 2015 to 2025.

Before chemical preparation and instrumental analysis, all raw tissue samples were thoroughly homogenized using an ultra-centrifugal mill with high-grade steel blades to prevent metal contamination (Retsch’s Grindomix Knife Mills (Haan, Germany), Verder International (Vleuten, The Netherlands)). After homogenization, approximately 0.5 g of tissue was weighed, 2 mL of ultrapure water was added, it was vortexed for a few seconds, then 3.5 mL of HNO_3_ (trace metal grade) was added. Mineralization was performed by advanced microwave-assisted acid digestion using an UltraWAVE system (Milestone, Sorisole, Italy) operating with advanced Single Reaction Chamber (SRC) technology capable of handling high-pressure organic digestions.

The microwave operating temperature program was meticulously optimized for complex biological tissues, executing a linear ramp to reach the target conditions over a 23-min heating phase at 1500 W (reaching up to 240 °C on sensor T1 and 60 °C on T2, at a maximum pressure of 140 bar), followed by an isothermal holding phase for 10 min to guarantee the complete oxidative destruction of all carbonaceous organic fractions, fully digest resistant biological structures, and prevent any volatile loss of target analytes.

After completion of the heating cycle and subsequent cooling, the resulting clear and transparent digestates were quantitatively transferred and diluted to exact final volumetric marks (50 mL volumetric flasks) with ultra-pure water.

### 2.3. Cadmium Analysis

To correct for instrumental drift, plasma fluctuations, and matrix suppression effects during high-throughput quantification, all analytical blanks, calibration standards, reference materials, and samples were spiked with Rhodium (Rh) as an internal standard. Elemental analysis was performed by inductively coupled plasma mass spectrometry using an Agilent 7800 ICP-MS instrument (Santa Clara, CA, USA) operating in helium (He) cell mode to effectively eliminate troublesome polyatomic interferences.

Instrumental analyses were performed using an Argon plasma sustained by a 1500 W RF generator. The sample introduction system was equipped with a Meinhard concentric nebulizer coupled with a quartz Scott-type double-pass spray chamber featuring Peltier-effect cooling. Mass spectrometer acquisition conditions utilized ultra-pure helium as the collision cell gas, maintained at a flow rate of 4.3 mL/min.

Method blanks, procedural spikes, and certified reference materials (CRMs), specifically DOLT-4 (Dogfish Liver, National Research Council Canada), DOLT-5 (Dogfish Liver, National Research Council Canada), and Bovine Liver (European Commission, JRC Reference Materials), were processed in triplicate alongside each analytical batch. Recovery rates for cadmium in all certified reference materials consistently ranged between 95% and 105%, confirming the accuracy, precision, and reliability of the established analytical procedure. The limit of detection (LOD) and limit of quantification (LOQ) for cadmium were 0.0006 mg/kg and 0.0020 mg/kg wet weight, respectively.

Statistical analyses and chemometric modelling were performed using the Chemometric Agile Tool (CAT), developed by R. Leardi, C. Melzi, and G. Polotti (freely available at http://gruppochemiometria.it/index.php/software, accessed on 2 February 2026). Data were standardized using mathematical autoscaling (mean-centring followed by division by the standard deviation of each individual variable) to eliminate scale discrepancies among different tissue matrices and subsequently subjected to PCA.

## 3. Results

Descriptive statistics of cadmium concentrations across the analysed tissues and species are summarized in Table 1. Equines exhibited significantly higher Cd concentrations in both liver (mean 10 mg/kg, median 8.5 mg/kg) and kidney (mean 53 mg/kg, median 48.2 mg/kg) compared to bovines and cervids, as confirmed by a chi-square test (*p* < 0.001). The renal burden in equines was particularly extreme, reaching a maximum value of 140 mg/kg, which was substantially higher than the values observed in any other species or tissue category.

Bovines and cervids displayed relatively low and comparable Cd levels in hepatic and renal tissues. Mean hepatic concentrations for bovines (0.14 mg/kg) and cervids (0.13 mg/kg) were nearly identical, while renal means were slightly higher in cervids (0.72 mg/kg) compared to bovines (0.60 mg/kg). Across all three species, Cd concentrations in muscle tissue remained extremely low, with mean values consistently below 0.10 mg/kg (0.022 mg/kg for bovines, 0.017 mg/kg for cervids, and 0.078 mg/kg for equines).

**Figure 1:**
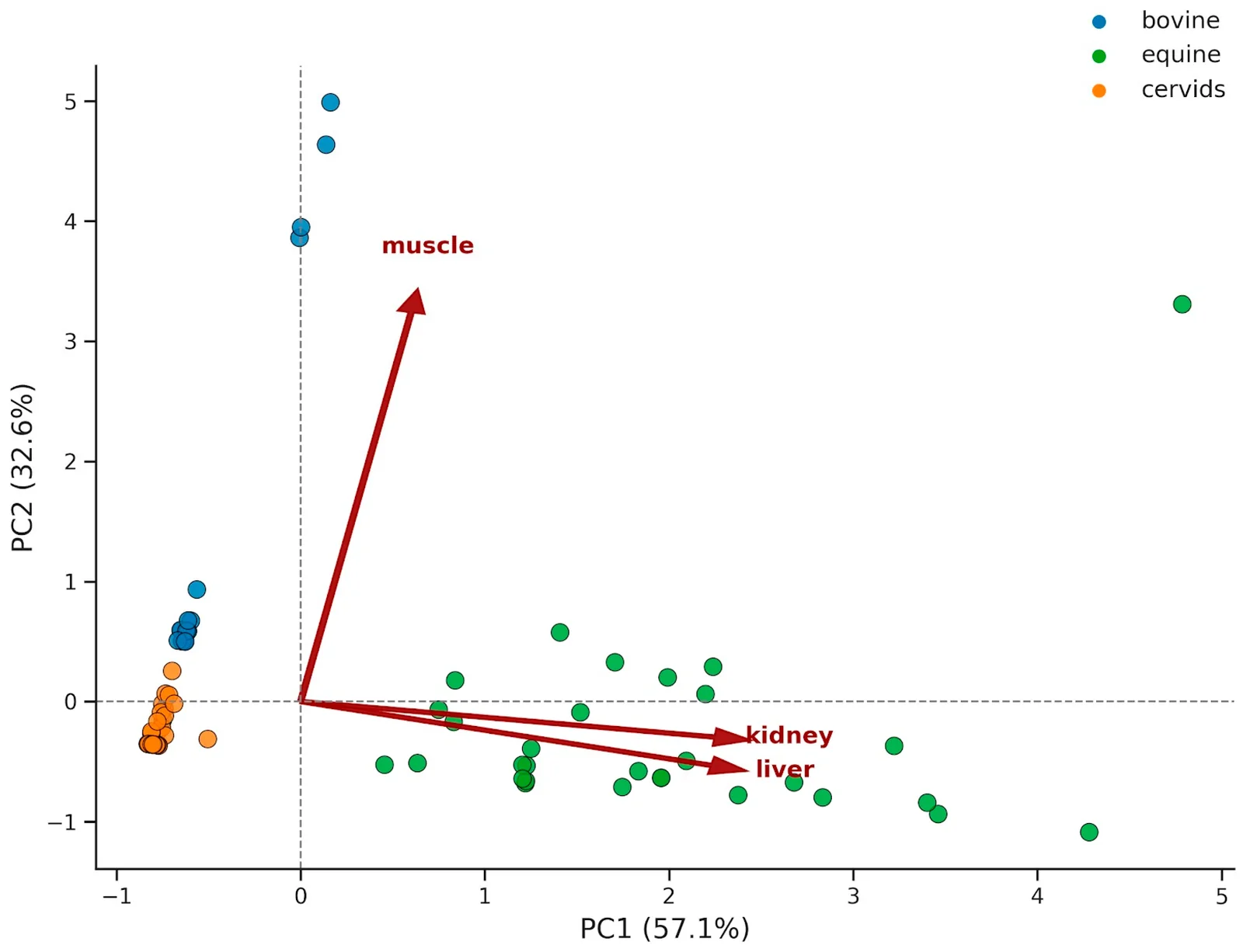
Principal Component Analysis biplot of cadmium concentrations in bovine, equine, and cervid tissues. The scores (points) represent individual animals, color-coded by species, while the vectors (red arrows) represent the loadings of the analysed tissues (liver, kidney, and muscle). The first two principal components (PC1 and PC2) account for 57.1% and 32.6% of the total variance, respectively. The separation along the PC1 axis indicates that hepatic and renal Cd burdens are the primary drivers distinguishing equines from the other two species.

The PCA biplot (Figure 1) illustrated distinct species-specific patterns of Cd accumulation across the study populations. A clear separation was observed along the PC1 axis (explaining 57.1% of the total variance), which is strongly correlated with both hepatic and renal Cd concentrations. Equines were widely dispersed along the positive side of the PC1 axis, reflecting significantly higher and more variable tissue burdens compared to bovines and cervids, which clustered tightly near the origin. Conversely, the muscle vector, showing a strong loading on the PC2 axis (accounting for 32.6% of the variance, bringing cumulative explained variance to 89.7%), confirmed that muscle tissue contributes minimally to the overall variance, consistent with its role as a secondary target compared to the primary accumulation sites of the liver and kidneys.

**Figure 2:**
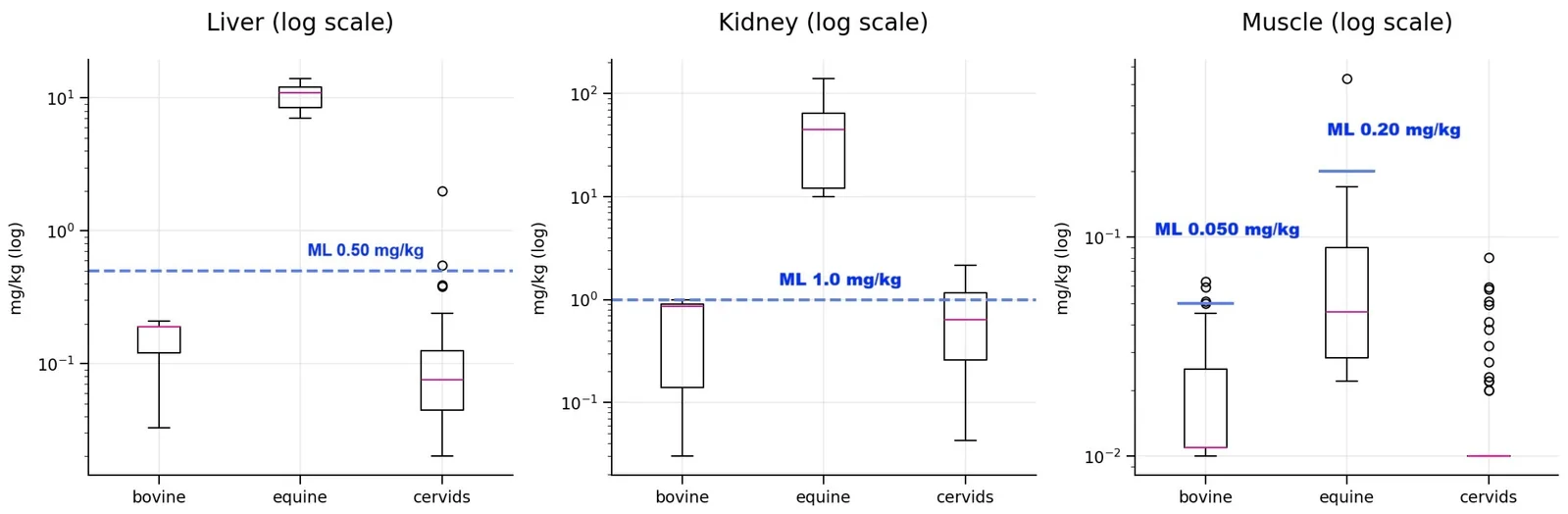
Distribution of cadmium concentrations (mg/kg wet weight) in liver, kidney, and muscle tissues across bovine, equine, and cervid species. Boxplots represent the median, interquartile range (IQR), and outliers of Cd concentrations on a logarithmic scale. Horizontal dashed/solid blue lines indicate the corresponding Maximum Levels (MLs) for cadmium set by Commission Regulation (EU) 2023/915: 0.50 mg/kg for liver, 1.0 mg/kg for kidney, and 0.050 mg/kg or 0.20 mg/kg for muscle (depending on the species). Note the systematic exceedance of regulatory limits in equine tissues compared to the compliant profiles observed in bovines and cervids.

The comparative analysis of cadmium tissue burdens against the Maximum Levels (MLs) established by Commission Regulation (EU) 2023/915 revealed significant implications for food safety assurance. As illustrated in Figure 2, the vast majority of bovine and cervid samples remained fully compliant with these regulatory thresholds. In contrast, the equine population consistently exhibited concentrations exceeding these limits, highlighting an urgent need for targeted safety assessments regarding equine-derived food products. Specifically, hepatic Cd burdens in the equine cohort systematically exceeded the 0.50 mg/kg threshold, while renal concentrations were orders of magnitude higher than the 1.0 mg/kg ML. However, only a minimal fraction of equine muscle samples (3.33%) exceeded the 0.20 mg/kg ML established for horsemeat.

## 4. Discussion

The multivariate chemometric analysis performed in this study highlighted significant differences in cadmium tissue distribution among the investigated ungulate species [11]. As shown in the PCA biplot, equines were widely dispersed along the positive side of the PC1 axis, reflecting significantly higher tissue burdens compared to the other groups. This suggests that while bovine and cervid exposure profiles remain relatively homogeneous and low-level, the recognized longevity and unique physiological capacity of equines lead to a more pronounced and heterogeneous bioaccumulation of Cd in metabolic organs. Furthermore, the clustering tightness observed for bovines reflected standardized industrial feeding rations, whereas the wider dispersion in cervids highlighted natural ecological foraging plasticity across diverse geoclimatic zones of the Aosta Valley, a small and predominantly mountainous region located in northwestern Italy (45°44′ N 19′ E) from which they originated. While this region shares a coherent macro-climate and uniform regional background conditions, there are localized micro-environmental variations within the alpine territory, such as differing altitudes, exposure aspects, and localized vegetation types across sub-valleys.

The high dispersion of equines can also be attributed to broader age ranges within commercial slaughter populations, differences in lifetime cumulative forage consumption rates, and exceptionally efficient renal reabsorption and hepatic sequestration mechanisms characteristic of perissodactyls. Conversely, the striking similarity between bovines and cervids near the origin of the PCA plot suggests that, in this studied environment, these species share relatively low and homogeneous exposure pathways, reflecting regional geochemical background levels, atmospheric deposition rates, and strictly regulated agricultural feeding regimes [6,8]. Our findings reinforce the hypothesis that equines represent a uniquely valuable bioindicator model for environmental Cd monitoring due to their higher capacity for sequestration in the liver and kidneys [9].

The minimal contribution of muscle tissue to the PC1 variance, confirmed by the strong loading of the muscle vector on the PC2 axis (accounting for 32.6% of the variance, bringing cumulative explained variance to 89.7%), demonstrates that muscle tissue contributes minimally to the overall variance, consistent with its role as a secondary target compared to the primary accumulation sites of the liver and kidneys. Consequently, these organs should remain the absolute focus of veterinary surveillance and food safety protocols, particularly in species-specific risk assessments, as generalized monitoring frameworks might severely underestimate the higher bioaccumulation potential of equines [7].

These findings underscore a critical food safety concern: while local bovine livestock and wild cervid populations appear to pose a negligible risk regarding dietary Cd exposure, the consumption of equine offal, and to a lesser extent, muscle tissue, likely originating from older individuals, requires much more stringent risk management, trace-back auditing, and ongoing surveillance [5,9]. However, in the context of official food safety controls and official maximum limits, these analysed samples represent the actual commercial products and harvested game directly consumed by the human population. Therefore, these findings provide a definitive, realistic assessment of real-world dietary cadmium exposure and the effective dietary burden entering the food chain, offering immediate evidence for food safety surveillance.

The fundamental anatomical and physiological divergences in digestive strategies between ruminant artiodactyls (bovines and cervids) and hindgut-fermenting perissodactyls (equines) should be considered. Ruminants use pre-gastric rumen fermentation, while selective browsers like red deer (*Cervus elaphus*) rely on multi-chambered forestomachs, slow fermentation, and foraging behavior to influence metal absorption. Equines use post-gastric hindgut fermentation and pair a trickle-feeding strategy with high dry matter intake to drive elevated lifetime metal uptake [12].

Following gastrointestinal absorption, the systemic transport and internal distribution of cadmium are governed by advanced cellular detoxification mechanisms. The combination of cadmium’s extreme systemic toxicity, its total lack of biological utility, and its progressive accumulation in long-lived species implies the presence of highly evolved cellular detoxification pathways [1,4]. These physiological mechanisms allow organisms to sequester cadmium, neutralizing its acute detrimental impacts and accommodating extended lifespans [10]. In fact, cattle are routinely slaughtered at a much younger age (typically under 24 to 30 months) compared to equines, which often experience substantially longer productive or working lifespans before entering the food chain. Because horses experience prolonged environmental exposure over extended lifespans, their physiological response involves the robust upregulation of metallothioneins, low-molecular-weight, cysteine-rich proteins localized primarily in the liver, which bind cadmium with exceptionally high thermodynamic stability, effectively preventing systemic cellular damage and shielding vital enzymes from xenobiotic inhibition [10].

Circulating cadmium-metallothionein complexes from the liver are filtered by kidney glomeruli and reabsorbed in proximal tubular cells [13]; lysosomal degradation releases free Cd ions that cause oxidative stress, mitochondrial dysfunction, cell necrosis, and nephropathy if thresholds are met [3,13]. Equines run this renal–hepatic recycling loop with high efficiency, turning their kidneys into a permanent sink for cadmium. This creates a unique biological paradox in long-lived ungulates: the very intracellular mechanisms that successfully neutralize and sequester cadmium to protect against acute systemic toxicity also result in massive, age-dependent bioaccumulation over time [10]. The physiological tendency of equines to sequester high levels of Cd in metabolic organs, coupled with their extended lifespans, frequently results in tissue burdens that dramatically exceed standard European Union contaminant limits for food-producing animals [14].

## 5. Study Limitations

As this study is based on data from an official analytical control laboratory, individual age or sex records for the sampled animals were not accessible. The lack of precise age ranges and sex information represents a key limitation, which should be factored into future interpretations, as these variables could potentially influence interspecific comparisons of cadmium accumulation. Moreover, the intrinsic variability of environmental and management factors, ranging from soil composition and fertilizer application rates to housing systems and regional atmospheric deposition, can introduce significant fluctuations into baseline metal concentrations. Future investigations should prioritize stratified sampling protocols that capture specific developmental stages, exact sex ratios, and standardized environmental metadata to isolate true toxicological mechanisms from field-level variables.

## 6. Conclusions

Cadmium accumulation exhibits marked species- and tissue-specific patterns across the investigated ungulates, with hepatic and renal tissues serving as reliable exposure biomarkers, while equines demonstrate dramatically higher metal burdens than bovines and cervids. Principal Component Analysis effectively captures these multidimensional toxicological structures, confirming that liver and kidney burdens are the primary factors separating equines from ruminants. This chemometric method serves as a robust analytical marker reflecting the distinct toxicokinetic dynamics governed by species physiology. The findings underscore the necessity of updating species-specific food safety guidelines and incorporating comparative tissue metal profiling into veterinary public health and environmental monitoring frameworks.

## Figures and Tables

**Table 1:** Cadmium concentration in animal tissues (mg/kg wet weight).

**LIVER (N = 90)**					**Regulation 915/2023 UE**
	Mean	Median	Min	Max	LM
**Bovine** (n = 30)	0.14	0.19	0.033	0.21	0.50
**Cervids** (n = 30)	0.13	0.076	0.020	2.0	
**Equine** (n = 30)	10	11	7.0	14	
**KIDNEY (N = 90)**					
	Mean	Median	Min	Max	LM
**Bovine** (n = 30)	0.60	0.86	0.030	1.0	1.0
**Cervids** (n = 30)	0.72	0.64	0.043	2.2	
**Equine** (n = 30)	53	45	10	140	
**MUSCLE (N = 90)**					
	Mean	Median	Min	Max	LM
**Bovine** (n = 30)	0.022	0.011	0.010	0.63	0.050
**Cervids** (n = 30)	0.017	0.010	0.010	0.081	
**Equine** (n = 30)	0.078	0.046	0.022	0.53	0.20

## Data Availability

The raw data supporting the conclusions of this article will be made available by the authors on request.
